# P37 Retrospective analysis of the microbiological structure of pathogens in severe lung lesions.

**DOI:** 10.1093/jacamr/dlaf118.044

**Published:** 2025-07-14

**Authors:** Oksana Viltsaniuk, Viktor Syvak, Nataliia Tkachuk, Dmytro Lushnikov, Oleksandr Viltsaniuk

**Affiliations:** National Pirogov Memorial Medical University, Vinnytsya, Ukraine; Military Medical Clinical Centre of the Central Region, Ukraine; Military Medical Clinical Centre of the Central Region, Ukraine; Military Medical Clinical Centre of the Central Region, Ukraine; National Pirogov Memorial Medical University, Vinnytsya, Ukraine

## Abstract

**Background:**

Combat-related infectious complications, including lung damage, remain an urgent clinical problem. The microbiological spectrum of pathogens changes under the influence of the nature of wounds, treatment conditions and the use of antimicrobial agents.

**Objectives:**

To analyse the dynamics of changes in the microbiological spectrum of pathogens in servicemen before and after the full-scale invasion of Ukraine by the Russian Federation during 2014–24.

**Materials and methods:**

The results of 219 microbiological studies of blood, sputum, pleural fluid and bronchoalveolar lavage in patients with severe injuries and lung lesions during 2014-2024 were analysed. These results were also divided by year according to the escalation of the military conflict in Ukraine, such as before 2014–22 (110 results) and after the full-scale invasion of 2022–24 (109 results). A total of 219 microbiological test results (blood, sputum, pleural fluid, and bronchoalveolar lavage) were analysed from patients with severe lung injuries. The data were divided into two periods: before the full-scale invasion (2014–22, *n*=110) and after (2022–24, *n*=109).

**Results:**

The analysis of the results of cultures for the period 2014-2022 revealed that out of 110 cultures, 96 (87%) were informative. Of these, 74% were inoculated as a monoculture and 13% in combination. The largest group of microorganisms isolated from patients with severe lung injury was dominated by a group of non-fermenting Gram-negative bacteria (47.2%), namely *Pseudomonas aeruginosa* 30.3% and *Acinetobacter baumannii* 15.7%. *Staphylococcus haemolyticus* was isolated in 13%, and pathogens such as *Staphylococcus aureus* 3.8%, enterococci 5.7%. Other pathogens were isolated in single cases and their total number was not clinically significant. In the analysis of cultures obtained from patients in 2022–24, 94 (86%) of 109 cultures were informative. In most cases, *Klebsiella pneumoniae* (43%) and *S. aureus* (21%) and *A. baumannii* (10%) were detected. In the same ratio *Enterobacter* 9% and *Proteus* 9%, *Bacillus* spp. 7%. In some cases, *Lelliottia amnigena*, *Staphylococcus epidermidis*, *S. haemolyticus*, *Staphylococcus lentus* were isolated (1%, respectively).

**Conclusions:**

During the analysed period, there were changes in the species composition of pathogens in patients with severe pulmonary pathology, which may be associated with changes in immune status, as well as a reflection of the transformation of the nature of hostilities and as a consequence of severe polytrauma.

